# Mechanical Treatment of Deformities

**Published:** 1865-08

**Authors:** C. R. Blackall

**Affiliations:** 79 Dearborn Street; Chicago, Ill.


### MECHANICAL TREATMENT OF DEFORMITIES.

By C. R. BLACKALL, M. D., of Chicago.

Many causes have combined in producing a variety of Deformities which are not amenable to any method of treatment within the scope of the general practitioner. Curvatures of the spine, distortion of the chest, enlargement of the joints, contractions and atrophy of the limbs, are among the number, and have become objects of frequent, if not daily observation, while very little is attempted toward effecting a cure, under the belief that nothing of a permanent character can bé accomplished. But it may be abundantly demonstrated that a large majority of such cases are susceptible of cure by mechanical means, if such means are properly and persistently applied.

A method of special practice, originating in Europe some fifty years since, but elaborated and improved through the genius and assiduity of a few American physicians, has become developed into a definite system, based upon and in harmony with physiological laws. Its varied measures are proven to be of such character as will so modify the processes of nutrition that a healthy, in place of a diseased, action will be induced. It has received a popular title which, for want of a better, will probably cling to it, being known as the "Movement Cure." Essentially, it is a series of exercises, of prescribed quality and quantity, administered at the hands of the physician and assistants, with the aid of ingenious apparatus, frequently devised expressly for the particular needs of the case in hand. These exercises, motions, or "movements," vary in character, according to the nature of the disease and the age or condition of the patient. They are equally applicable to childhood and old age, because their degree can be easily adapted to either. In general, it is necessary that attention should be bestowed upon each case every day, and with as much regularity as possible. In its administration, the tact and skill gained alone by daily experience, added to necessary professional attainments, are essential, in order to secure success, because, not only is the muscular effort to be made or induced, but the mental state must be so controlled, that a thorough consentaneousness of action will result. The physician who fails in this latter point must necessarily fail in all, whether in general or special practice, but especially in this, where weeks must often elapse ere the results, perfectly certain to the operator, can be satisfactorily demonstrated to the superficial, though anxiously interested observer, or even to the patient himself.

I need not, perhaps, be more explicit in the matter of details, yet it is evident that such a system cannot be carried out except some specified time and place be devoted to it. Under such conditions the results attained will prove it a valuable agency in the curing of disease, and one that once understood will be fully appreciated by the medical profession, as well as by the large class of sufferers whom it proposes to relieve.

We are frequently under a necessity for prescribing EXERCISE for our patients, indeed, very often it is the only thing necessary in order to secure return of Health, but how, when and in what degree, it shall be used, are not easy to direct, and we are left to a few general principles, the whole matter being subject rather to the judgment of the patient than of the physician, hence the frequent failures in this direction. Yet the exercise must be obtained in some way, if the patient ever fully recovers. But it is not practicable for the general practitioner to stop, in each case, and administer that which may be urgently needed, because of the numerous demands upon his time, as well as for other reasons. Here, then, occurs the need of a specialty, and the result is found in a system which does not assume to be a universal panacea, but really is a valuable adjunct, if not certain means, in very many instances, and one which leading members of the profession not only pronounce rational, but eminently valuable, and successful.

I beg to submit a few recent cases, by way of illustration: Mrs. ——, under the medical care of a distinguished gentleman of this city, was brought to me by her physician for treatment. The right arm and hand were much atrophied, and the wrist and hand in a state of rigidity, in consequence of Inflammatory Rheumatism. Within two months the use less members were fully restored, both as regards size and the mobility of the joint.

Miss ——, in a condition of great general debility, and with well-marked lateral curvature of the spine, became my patient in April, 1864. She returned home in July following, relieved of her deformity, and able to resume her duties as a teacher.

Master Willie ——, aged three years. Angular curvature of the spine, in the cervical region. Head drawn sharply backward. Very little motion, in any direction, of the head upon its axis. Extreme sensitiveness of the whole nervous system. Three months time sufficed to produce an entire cure.

Miss ——, aged eight years. Right leg atrophied, ankle joint immovable. Commenced in April last, and is still under treatment. No longer needs any artificial support to the limb, which is growing finely and strong, and every indication present of a speedy and permanent cure.

I have given these few cases out of many, with the hope of calling the attention of the profession to what can be done for this large class of unfortunates. It is not possible to intelligently describe the peculiar processes adopted in each, but I shall be happy to give all desired information, on personal application, or by letter, if practicable.

79 DEARBORN STREET.

